# Self-Esteem and Feelings of Inferiority and Superiority Among Athletes and Non-Athletes

**DOI:** 10.3390/ejihpe15020022

**Published:** 2025-02-08

**Authors:** Stanislava Stoyanova, Nikolay Ivantchev

**Affiliations:** Department of Psychology, South-West University “Neofit Rilski”, 2700 Blagoevgrad, Bulgaria; nikyart@swu.bg

**Keywords:** inferiority feelings, self-esteem, superiority feelings

## Abstract

Self-esteem is a central part of personality, related to self-perceptions and evaluation of oneself compared to other people. Self-esteem could be global self-esteem, concerning the whole personality, or partial self-esteem, concerning the different aspects of personality and performance. Global self-esteem, as well as feelings of inferiority and supremacy, were compared among 197 athletes and 198 non-athletes in Bulgaria based on three self-reported questionnaires—the inferiority complex shortened scale COMPIN-10, the superiority complex shortened scale SUCOMP-10, and the single-item self-esteem scale. It was found that the athletes had significantly higher self-esteem and more strongly expressed feelings of superiority, as well as fewer experienced feelings of inferiority than the non-athletes. This may be due to athletes’ achievements and their recognition by society, as well as the social image imposed by media as rich, successful, and representatives of their country. High self-esteem is necessary for athletes to be confident in their ability to perform well during sports competitions. More years of sport experience correlated with a decrease in inferiority feelings and an increase in self-esteem. There were not any statistically significant differences between the athletes practicing individual sports and the athletes practicing team sports in their inferiority feelings, superiority feelings, or self-esteem.

## 1. Introduction

It is important to find some socially appropriate ways to reduce inferiority feelings and increase self-esteem and superiority feelings because they can affect quality of life and life satisfaction. Inferiority feelings can limit daily life activities (Hirao, 2014), which is less probable for athletes, who presumably live a more active life than non-athletes. It is important to reduce feelings of inferiority in everyday life (Hirao, 2014) to reduce the negative feelings of shame, inadequacy, and rejection and to increase self-esteem (Lamberson & Wester, 2018). This paper aims to establish if physical activity and sport could be an effective way to reduce inferiority feelings and increase self-esteem and superiority feelings by comparing global self-esteem, as well as feelings of inferiority and supremacy among two groups of athletes and non-athletes of a similar age.

### 1.1. Self-Esteem

Self-esteem is a central part of personality, related to self-perceptions and evaluation of oneself compared to other people. Self-esteem includes the sense of being loved, the sense of being capable (Pimentel et al., 2018); the sense of self-worth (Lamberson & Wester, 2018; Liu, 2022; Pimentel et al., 2018); a positive emotional experience based on self-image; evaluation of one’s degree of respect, significance, importance, success, or competence; and understanding of one’s social value (Liu, 2022; Pimentel et al., 2018) depending on a person’s life experiences, for example, feedback from social interaction (Brailovskaia & Margraf, 2020), recognition of skills or inabilities, success or failure (Pimentel et al., 2018). Self-esteem could be global self-esteem concerning the emotional evaluation of the whole personality (own behavior and characteristics), reflecting the attitudes toward the self as self-acceptance or self-rejection (Brailovskaia & Margraf, 2020; Pimentel et al., 2018; Robins et al., 2001), as well as partial self-esteem concerning the domain-specific aspects of personality and performance (Brailovskaia & Margraf, 2020; Robins et al., 2001). Of course, global self-esteem correlates positively with domain-specific self-evaluations, for example, those concerning academic outcomes, psychological and physical health, self-assessed athletic abilities, etc. (Robins et al., 2001).

Elite athletes have higher self-esteem (global and partial self-esteem—related to mind, ability, authority, and confidence) than non-elite athletes (Petrovska et al., 2022). Self-esteem is higher among national athletes compared to regional athletes (Saha et al., 2024). Self-esteem is also higher among successful athletes who gain medals in regional, national, and international competitions than among non-successful athletes who do not manage to gain medals (Matmask & Ozrudi, 2019). Practicing sport from an earlier period in life is related to higher self-esteem, as students who participated in sport prior to their enrollment in a university or college had higher self-esteem and were happier than students who did not practice sport prior to their university studies (Collins, 2018).

Low self-esteem could result from a perceived incongruence between the self-concept (who one believes oneself to be) and the self-ideal (who one believes he or she should be), which increases feelings of incompetence and inferiority (Lamberson & Wester, 2018). This incongruence often results from social comparisons that affect feelings of inferiority, and those who view themselves as less than others often have low self-esteem (Lamberson & Wester, 2018).

### 1.2. Feelings of Inferiority

Inferiority has been defined in the scientific literature both as a composite feeling (Adler, 2007; Čekrlija et al., 2017; Lamberson & Wester, 2018; Rokvic, 2020; Sweeney, 2009; D. Yang et al., 2023) and as a cognitive appraisal (Liu, 2022; Rokvic, 2020). Inferiority represents a mixture of feelings of the brevity of existence (Adler, 2007), insecurity (Adler, 2007; D. Yang et al., 2023), imperfection (Adler, 2007), incompleteness (Sweeney, 2009), impotence (Rokvic, 2020), powerlessness (D. Yang et al., 2023), incompetence (Čekrlija et al., 2017), dependence on others (Čekrlija et al., 2017; Lamberson & Wester, 2018; Rokvic, 2020; Sweeney, 2009), worthlessness (Lamberson & Wester, 2018), disappointment (D. Yang et al., 2023), inadequacy, shame (Lamberson & Wester, 2018), an emotional experience of rejecting the self (Liu, 2022), feeling not being accepted by others and having a low sense of belongingness (Lamberson & Wester, 2018) accompanied with a negative subjective evaluation of oneself (Lamberson & Wester, 2018; Liu, 2022; Rokvic, 2020) as not as good as others, not able to succeed (Adeka, 2019), not able to achieve a goal (D. Yang et al., 2023), failing (Lamberson & Wester, 2018), being less capable (Čekrlija et al., 2017), and lack of certainty in one’s abilities and competence (Lamberson & Wester, 2018). Low self-esteem and decreased self-efficacy accompany feelings of inferiority (Lamberson & Wester, 2018). Inferiority feelings are significantly associated with depression (Hirao, 2014; Lamberson & Wester, 2018), suicidal ideation, frustration (Hirao, 2014), envy (Lamberson & Wester, 2018), insomnia, sleep disorders, and pain (Hirao, 2014).

### 1.3. Origin of Inferiority

A child’s perception of oneself as smaller and weaker than adults (Lamberson & Wester, 2018), who are stronger (Adeka, 2019) and possess such power that struggling against this power is hopeless (Čekrlija et al., 2017), can trigger a sense of inferiority among children (Lamberson & Wester, 2018) caused by negative experience (Adeka, 2019; Liu, 2022). Feelings of inferiority can result from negative social comparisons (Lamberson & Wester, 2018), provoking unfavorable comments by the environment (Rokvic, 2020), giving the impression that the world is hostile (Adler, 2007). As soon as a child encounters greater difficulties (Adler, 2007) or physical, psychological, or social obstacles (D. Yang et al., 2023), the impression of hostility is reinforced, which often occurs in children with disabilities (Adler, 2007) or defects in appearance, such as weight issues, visual defects, skin diseases, burn wounds, speech defects (Adeka, 2019), etc.

### 1.4. Inferiority Complex

There are varying degrees of inferiority (D. Yang et al., 2023). When feelings of inferiority become overwhelming, an inferiority complex emerges (Sweeney, 2009). The highest degree of inferiority is labelled as an inferiority complex. The symptoms of an inferiority complex are social withdrawal because of fear of failure (Adeka, 2019; D. Yang et al., 2023); fear of stereotyping and discrimination; feeling disrespected, angry (Adeka, 2019), discouraged (D. Yang et al., 2023), and unable to fit in with a group or a task (Adeka, 2019); and feelings of self-doubt (Adeka, 2019; Lamberson & Wester, 2018; D. Yang et al., 2023), low self-esteem (Adeka, 2019; Čekrlija et al., 2017; Lamberson & Wester, 2018), and low self-confidence (Adeka, 2019; D. Yang et al., 2023).

An inferiority complex can lead to some unhealthy ways of life (Sweeney, 2009) with such symptoms of illness (Sweeney, 2009) like a neurotic disposition of the individual (Rokvic, 2020; D. Yang et al., 2023), depression (D. Yang et al., 2023), and anxiety (Čekrlija et al., 2017; D. Yang et al., 2023). An inferiority complex can lead to general misanthropy as a lifestyle, an unfavorable attitude towards any challenge (Čekrlija et al., 2017), a useless life impeding one’s own psychological growth (Sweeney, 2009; D. Yang et al., 2023), being prone to addictions, fantasies (D. Yang et al., 2023), justifications, alibis (Čekrlija et al., 2017), and safeguarding mechanisms (Sweeney, 2009). Inferiority complexes are related to preference for the defense mechanisms regression and rationalization (Čekrlija et al., 2017).

An inferiority complex is characterized by persistent and excessive feelings of inferiority that are hard to be overcome (Adeka, 2019), but the inferiority complex can be compensated (D. Yang et al., 2023). Inferiority feelings lead to a striving to compensate and overcome inferiority. Inferiority feelings can manifest as abnormal when individuals do not use them as inspiration to achieve success (Lamberson & Wester, 2018).

### 1.5. Compensation of Inferiority Feelings—From Striving to Overcome Inferiority to Feelings of Superiority

Becoming aware of one’s inferiority stimulates a way to be compensated for it (Adler, 2007). Individuals recognize from a young age their own deficiencies (Lamberson & Wester, 2018). Already in the first days of childhood, children strive to attract the attention of parents to themselves (Adler, 2007). These are the first signs of the awakened striving for significance, which develops in conjunction with the sense of inferiority and makes the child set a goal that may make the child feel elevated above their own environment (Adler, 2007). The feeling of inferiority is what forces us to set goals in life and to reach them (Adler, 2007; Lamberson & Wester, 2018).

Feelings of inferiority are motivating (Lamberson & Wester, 2018). These feelings can motivate individuals to strive toward superiority, for example, by means of higher positions (Lamberson & Wester, 2018) compared to the others in the social hierarchy. People with the goal of superiority strive to be the best at everything, to achieve the highest academic grades, to win honors and high approval from others (Sweeney, 2009), and to become good students or athletes (Adler, 2008). It is especially important to foster the courage to be imperfect so that people striving for super achievement are not crushed by failures or defeats (Sweeney, 2009).

Feelings of inadequacy and the activity for compensatory superiority are normal when one understands and accepts the reality of the human condition (Sweeney, 2009). Many individuals and groups strive toward goals of superiority, power, wealth, or position (Sweeney, 2009). However, we should avoid the great emphasis in our society on competition and the manifested signs of superiority (Sweeney, 2009).

The way in which the individual will strive to achieve superiority is determined by the magnitude of the sense of community and social interest (Adler, 2007). High-social-interest persons may work very hard to achieve success, enjoying the interactions with other people and not doing harm to them (Sweeney, 2009). Attention to enjoyment, satisfaction, and sharing with others can refocus individuals’ interest toward what they are doing rather than how they are doing it (Sweeney, 2009).

Low-social-interest individuals are not interested in maintaining good relationships with others (Sweeney, 2009), striving for power and superiority over others (Adler, 2007) by means of uncooperative behavior (Sweeney, 2009), aggression (Rokvic, 2020), and hostility (Hirao, 2014). Some family beliefs, such as “I am worthwhile and belong only when I am the best,” are related to the faulty goal of superiority expressed, for example, as super striving for the best grades, most honors, being first in the class, etc. (Sweeney, 2009), which is not possible under all conditions and situations. Some corrective methods in the case of an extreme goal of superiority are avoiding blanket approval, encouraging the courage to be imperfect, and encouraging social cooperation (Sweeney, 2009).

The positive or negative effect of inferiority feelings on individual development depends on the compensation attitude of the individual (D. Yang et al., 2023). The inferiority compensation could be realized as self-compensation and other-compensation (D. Yang et al., 2023). Self-compensation is when the perpetrator of compensatory behavior is only the self and no others (D. Yang et al., 2023). Self-compensation means that the individual takes the initiative to overcome their own shortcomings and puts in some effort relying on their own strengths in order to improve their own abilities in a certain area and to succeed (D. Yang et al., 2023).

Compensation behavior could be completed by the self or by others (D. Yang et al., 2023). Other-compensation means that the individual feels self-confidence boosting indirectly brought about by the success or honor of their team and relational others (D. Yang et al., 2023), which is facilitated in sport. There is more possibility for other-compensation to occur in a collectivist culture than in an individualist culture (D. Yang et al., 2023).

A person could pursue a sense of superiority without any attempts to change the surrounding environment (D. Yang et al., 2023) or oneself. If the individual improves the environment or oneself motivated by their own striving to overcome the feelings of inferiority, then compensation is effective, and inferiority plays a positive role (D. Yang et al., 2023).

Inferiority feelings are important to enhance the growth of individuals (Hirao, 2014). The inferiority feelings activate compensatory processes that make people want to improve, grow, and overcome their perceived weakness (Čekrlija et al., 2017; Kolisnyk et al., 2020; Rokvic, 2020). Individuals often move toward mastery and competence in compensation for the feelings of inferiority (Sweeney, 2009).

### 1.6. Overcoming Feelings of Inferiority Through Sport

People can work to overcome the feelings of inferiority through leisure activities (Sweeney, 2009), for example, sports. There is evidence that through tennis, it is possible to transform one’s own feelings of inferiority into feelings of competence and competitiveness (Sweeney, 2009). Sport activity for 12 weeks has a significant positive correlation with the reduction in students’ inferiority complex (Liu, 2022). The competitive sports situation is better than the leisure sports situation in terms of the influence on students’ inferiority complex (Liu, 2022).

Physical exercise can directly and negatively predict inferiority feelings (T. Yang et al., 2023). Physical exercise can also indirectly predict inferiority feelings through the mediation of self-depletion and self-efficacy (T. Yang et al., 2023). Reducing self-depletion and improving self-efficacy are important ways to prevent feelings of inferiority (T. Yang et al., 2023). Inferiority feeling is more related to self-esteem and shame than to general self-efficacy (Lamberson & Wester, 2018).

Comparison to other athletes is an integral part of sports performance (Diel et al., 2021). Upward comparison is associated with feelings of shame and increased motivation, while downward comparison is related to happiness and lower levels of performance (Diel et al., 2021).

Pride in sport could be related to athletic self-identity, superiority, success in sports performance, and mastery (Kondo et al., 2022). Self-esteem correlates positively with pride from being successful and achieving (Kondo et al., 2022). Inferiority feelings concern physical fitness, appearance, self-esteem (D. Yang et al., 2023), poor performance in sports, and lack of some skills (Hirao, 2014).

### 1.7. Superiority Feelings and Superiority Complex

People strive to obtain a sense of superiority to compensate for their inferiority and change their situation (D. Yang et al., 2023). The feeling of superiority consists of ambitions (Lamberson & Wester, 2018), pride (Adeka, 2019; Kondo et al., 2022), self-perception of one’s own strengths (Adeka, 2019), personal supremacy (Adeka, 2019; Čekrlija et al., 2017), competence (Kondo et al., 2022), self-confidence (Čekrlija et al., 2017), achievement, self-efficacy, and assertiveness (Adeka, 2019).

Superiority is expressed in social identity (D. Yang et al., 2023), athletic self-identity, and self-esteem (Kondo et al., 2022). The feelings of superiority in sport could be related to the perception of oneself as having good physical fitness and mobility, achieving good results as a player, being successful, and being a leader of a team (Kondo et al., 2022).

An average person’s striving to be superior is manifested in the ambition to be successful, and if this person puts in efforts to succeed (Adeka, 2019) with endurance, diligence, and grit (Kondo et al., 2022), it does not lead to false valuations (Adeka, 2019).

A superiority complex develops when a person has an overly high opinion of oneself, accompanied with a feeling of omnipotence, competitiveness (Čekrlija et al., 2017), craving for attention, haughtiness, and arrogance, considering oneself as more important than others and one’s own opinion as better than others’ opinions (Adeka, 2019). That means that a superiority complex is related to narcissism. Mostly, narcissistic people know how to present themselves to gain attention and admiration, which increases their self-esteem (Brailovskaia & Margraf, 2020).

A person with a superiority complex decides to show others his or her own supremacy, manifesting as, for example, expensive material possessions or an obsession with vanity and appearances, as well as lacking feelings of adequacy (Adeka, 2019). A superiority complex is not due to authoritarianism (Čekrlija et al., 2017), alexithymia, or disgust to some stimuli (Rokvic, 2020).

It has been found that people who occupy high positions in an organizational hierarchy have a lower inferiority complex and higher superiority complex (Kolisnyk et al., 2020). An inferiority complex correlates negatively but weakly with a superiority complex (Rokvic, 2020).

### 1.8. Hypothesis

**H1.**  *Athletes demonstrate higher self-esteem compared to non-athletes.*

**H2.**  *Athletes demonstrate more strongly expressed feelings of superiority compared to non-athletes.*

**H3.**  *Athletes demonstrate fewer experienced feelings of inferiority compared to non-athletes.*

It was expected that the athletes would have higher self-esteem and more strongly expressed feelings of superiority, as well as fewer experienced feelings of inferiority than the non-athletes. This may be due to athletes’ achievements and their recognition by society, as well as athletes’ social image imposed by the media as rich, successful, and representatives of their country. High self-esteem is necessary for athletes to be confident in their ability to perform well during sports competitions. Furthermore, it has been found that self-esteem and general self-efficacy are enhanced during physical activity and decreased in the presence of an inferiority complex (Liu, 2022).

## 2. Materials and Methods

### 2.1. Procedure

Research was conducted from November 2023 to May 2024, both online using Microsoft Form (57% of the participants) and face-to-face in the form of a paper-and-pencil questionnaire (43% of the sample). There are some scientific findings that data collected from face-to-face and internet surveys may not differ only based on data collection method—face-to-face or online (Saloniki et al., 2019). The margin of error was the same—4.88, for both 57% and 43% of out 395 participants (*Sample size calculator*, n.d.)—so this difference of 14% should not significantly imbalance the responses.

The criteria for inclusion were being at least 18 years old, and the athletes were the target sample. Because all participants were adult and had studied physical exercises at school, as well as some of them possibly having practiced additional sport during their childhood or participated casually in sports activities, for the goals of this study, an athlete was considered an individual who was a member of a sport club, and/or studied a sport major in a university, or had a document of sport qualification, and/or practiced sport systematically at the moment of conducting this study. Systematical practice of sport was considered as practicing sport at least three times per week (Liutsko et al., 2024; Uçan & Çağlayan, 2012) since a minimum one year (Till et al., 2022). Additionally, training less frequently than twice per week does not change the muscle growth of the major muscle groups (Schoenfeld et al., 2016).

The athletes were approached with the cooperation of their coaches in different sports clubs and universities offering sport education. A part of the studied non-athletes were approached by means of their educators in some Bulgarian universities. All participants gave their informed consent and participated voluntarily, following the principles of the Declaration of Helsinki (World Medical Association, 2024).

### 2.2. Sample

More subjects were approached (*N* = 468), but only those with no missing answers were included in the sample. The sample consisted of 395 participants from 18 to 38 years old, almost equally distributed into athletes (49.9%) and non-athletes (50.1%)—see Table 1.

The athletes practiced individual sports (athletics, swimming, tennis, gymnastics, triathlon, skiing, karate, judo, etc.; *N* = 128) or team sports (baseball, football, basketball, handball, volleyball, field hockey, etc.; *N* = 69), according to the classification by Kondo et al. (2022).

### 2.3. Instruments

All questionnaires were translated into Bulgarian by a group of two psychologists and one philologist choosing the best translation suggested for the statements, their answers, and the instruction. Then, the items were translated back into English by another group of translators, and their translation was checked to correspond to the original questionnaires. This procedure followed the practices of translating questionnaires described by Przepiórkowska (2016). The questionnaires in Bulgarian can be found in Appendix A. A pilot study was conducted with 5 volunteers to establish how they understood the items of the questionnaires and to seek their further improvement.

#### 2.3.1. COMPIN-10 and SUCOMP-10

The inferiority complex scale COMPIN and the superiority complex scale SUCOMP have both full versions (with 40 items for inferiority complex and 38 items for superiority complex) and shortened versions (10 items in each scale) (Čekrlija et al., 2017) answered on a 5-point Likert scale (Čekrlija et al., 2020). The psychometric properties of their full versions were tested by the authors of these scales among 395 students (Čekrlija et al., 2017). The psychometric properties of their shortened versions were tested by the authors of these scales among 187 students between 19 and 41 years old (Čekrlija et al., 2017). The short versions of these scales, COMPIN-10 and SUCOMP-10, were used in the present study.

Cronbach’s α was 0.90 for COMPIN-10 in Bosnia and Herzegovina (Čekrlija et al., 2017; Čekrlija et al., 2020) and in Serbia (Rokvic, 2020), 0.89 in Malaysia, and 0.82 in India (Čekrlija et al., 2020).

Cronbach’s α was 0.88 for SUCOMP-10 in Bosnia and Herzegovina (Čekrlija et al., 2017; Čekrlija et al., 2020) and 0.86 in Serbia (Rokvic, 2020).

In the present study, Cronbach’s α was 0.88 and mean inter-item correlation was 0.435 for COMPIN-10, and Cronbach’s α was 0.82 and mean inter-item correlation was 0.317 for SUCOMP-10.

Overall sum score represents the individual’s measure of the inferiority feelings in the case of COMPIN-10 and the superiority feelings in the case of SUCOMP-10 (Čekrlija et al., 2020). The scores on each scale are averaged, dividing them by the number of the items in the scale (Rokvic, 2020).

#### 2.3.2. SISES

The single-item self-esteem scale (SISES) was created by Robins et al. (2001) and consists of the only item, “I have high self-esteem”, answered on a 5-point scale ranging from 1 (not very true of me) to 5 (very true of me) (Robins et al., 2001). The single-item self-esteem scale measures global self-esteem (Brailovskaia & Margraf, 2020). SISES is used primarily with the original 5 response options, but the response anchors could also vary from strongly disagree to strongly agree (Pegler et al., 2019). SISES could be answered also on a 7-point Likert-type scale ranging from 1 = “Not very common for me” to 7 = “Very common for me” (Pimentel et al., 2018), as it was used in the present study. The mean test-retest reliability estimate for the SISES is 0.75 (Robins et al., 2001).

#### 2.3.3. Sociodemographic Survey

Some sociodemographic data were also collected from the participants in this study regarding gender, age, years of sport experience, and the type of practiced sport. Answering the question “How long have you been practicing sport?”, the non-athletes reported that they did not practice sport. Correspondingly, the non-athletes also did not indicate any type of sport practiced by them. Additionally, the participants confirmed or rejected that they had practiced sport at least three times per week since a minimum one year. The athletes wrote the years of their sport practice and the type of sport that they practiced.

### 2.4. Data Analysis

Data were processed by means of SPSS 23 using descriptive statistics, independent samples *t*-test, two-way ANOVA, Pearson correlation coefficient, and binomial logistic regression.

## 3. Results

The years of sport experience and the scores on the inferiority scale, superiority scale, and single-item self-esteem measure were approximately normally distributed (see Table 2), as in several other countries the index of skewness suggested no significant distortion from normal distribution (Čekrlija et al., 2020). The distribution of age differed from the normal distribution (see Table 2).

**Figure 1:**
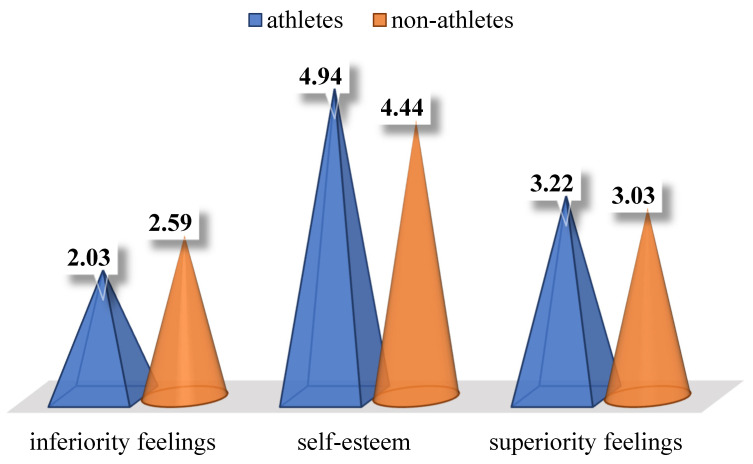
Averaged mean scores of athletes and non-athletes on inferiority feelings, global self-esteem, and superiority feelings.

The studied athletes (*N* = 197; *M* = 4.94; *SD* = 1.38) had significantly higher self-esteem (*F*_Levene_ = 11.606, *p*_Levene_ = 0.001; *t*_(385.277)_ = 3.316, *p* = 0.001, Cohen’s *d* = 0.334, i.e., small effect size, according to Lenhard and Lenhard (2022); see Figure 1) than the studied non-athletes (*N* = 198; *M* = 4.44; *SD* = 1.60).

The studied non-athletes (*N* = 198; *M* = 2.59; *SD* = 0.91) had significantly higher inferiority feelings (*F*_Levene_ = 11.037, *p*_Levene_ = 0.001; *t*_(378.238)_ = 6.782, *p* < 0.001, Cohen’s *d* = 0.682, i.e., intermediate effect size, according to Lenhard and Lenhard (2022); see Figure 1) than the studied athletes (*N* = 197; *M* = 2.03; *SD* = 0.74).

The studied athletes (*N* = 197; *M* = 3.22; *SD* = 0.73) had significantly higher superiority feelings (*t*_(393)_ = 2.665, *p* = 0.008, Cohen’s *d* = 0.268, i.e., small effect size, according to Lenhard and Lenhard (2022); see Figure 1) than the studied non-athletes (*N* = 198; *M* = 3.03; *SD* = 0.71).

**Figure 2:**
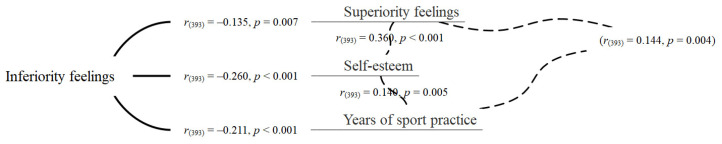
Correlations between the feelings of inferiority, superiority, self-esteem, and years of sport practice.

The increase in inferiority feelings correlated with a small decrease in superiority feelings, slightly higher self-esteem, and fewer years of sport experience (see Figure 2). Additionally, the increase in superiority feelings correlated with a medium increase in self-esteem and more years of sport practice (see Figure 2). More years of sport experience correlated with a small decrease in inferiority feelings, a small increase in superiority feelings, and slightly increased self-esteem (see Figure 2).

The non-athletes had significantly higher inferiority feelings than the athletes practicing individual sports (*p* Games–Howell < 0.001, see Table 3) and the athletes practicing team sports (*p* Games–Howell < 0.001, see Table 3). There were not any statistically significant differences between the athletes practicing individual sports and the athletes practicing team sports in their inferiority feelings (*p* Games–Howell = 0.834, see Table 3).

The athletes practicing individual sports had significantly higher superiority feelings than the non-athletes (*p* LSD = 0.028, see Table 3). The athletes practicing team sports had significantly higher superiority feelings than the non-athletes (*p* LSD = 0.033, see Table 3). There were not any statistically significant differences between the athletes practicing individual sports and the athletes practicing team sports in their superiority feelings (*p* LSD = 0.742, see Table 3).

The athletes practicing individual sports had significantly higher self-esteem than the non-athletes (*p* Games–Howell = 0.026, see Table 3). The athletes practicing team sports had significantly higher self-esteem than the non-athletes (*p* Games–Howell = 0.008, see Table 3). There were not any statistically significant differences between the athletes practicing individual sports and the athletes practicing team sports in their self-esteem (*p* Games–Howell = 0.652, see Table 3).

A binomial logistic regression was performed with the dependent variable being an athlete (coded with 1) or non-athlete (coded with 0) and the independent variables being inferiority feelings (the scores on COMPIN-10), superiority feelings (the scores in SUCOMP-10), global self-esteem (the scores on SISES), and age. There was not any multicollinearity between the independent variables, as tolerance was above 0.1 for all independent variables (0.640 for inferiority feelings, 0.781 for superiority feelings, 0.534 for global self-esteem, and 0.984 for age), and VIF was below 10 for all independent variables (1.562 for inferiority feelings, 1.281 for superiority feelings, 1.874 for global self-esteem, and 1.016 for age). There were not any extreme outliers in the data. The model was acceptable (for the omnibus test χ^2^_(3)_ = 90.759, *p* < 0.001, indicating that the independent variables explained the dependent variable well; for the Hosmer–Lemeshow test χ^2^_(8)_ = 7.841, *p* = 0.449, indicating that the independent variables did not explain the dependent variable badly). The independent variables explained about 27% of the variation in the dependent variable (Nagelkerke *R*^2^ = 0.274). Inferiority feelings explained statistically significant practicing of sport or not (Wald = 27.981, *df* = 1, *p* < 0.001). Superiority feelings did not explain statistically significant practicing of sport or not (Wald = 1.214, *df* = 1, *p* = 0.271). Global self-esteem did not explain statistically significant practicing of sport or not (Wald = 0.304, *df* = 1, *p* = 0.581). Age explained statistically significant practicing of sport or not (Wald = 31.626, *df* = 1, *p* < 0.001).

The chance that a person with more inferiority feelings was an athlete was 41.1% lower than for a person with fewer inferiority feelings (*b* = −0.888, Exp(*B*) = 0.411). The probability of being an athlete for a person with more inferiority feelings was 0.411/(1 + 0.411) = 0.291, i.e., 29.1%. The probability of being an athlete for a person with fewer inferiority feelings was 1 − 0.291 = 0.709, i.e., 70.9%.

The chance that an older person was an athlete was 84.6% lower than for a younger person (*b* = -0.167, Exp(*B*) = 0.846). The probability of being an athlete for an older person was 0.846/(1 + 0.846) = 0.458, i.e., 45.8%. The probability of being an athlete for a younger person was 1 − 0.458 = 0.542, i.e., 54.2%.

The person with the greatest chances of being an athlete was a younger person with fewer feelings of inferiority.

From the total number of athletes (*N* = 197), 76.1% (*N* = 150) were correctly classified, and from the total number of non-athletes (*N* = 198), 61.1% (*N* = 121) were correctly classified, so the total percentage of correct classification of subjects was 68.6%, which was an acceptable prediction as being higher than 50%, according to Anastasiei (2015).

## 4. Discussion

To summarize, it was found that the athletes had significantly higher self-esteem and more strongly expressed feelings of superiority, as well as less experienced feelings of inferiority than the non-athletes. This may be due to athletes’ achievements and their recognition by society, which are sources of pride and superior social comparisons. This may also be due to the athletes’ social image imposed by the media as rich, successful, and representatives of their country. Athletes’ superiority may be stimulated by their achievements, endurance, strength, and speed (Kisyov, 2021; Maderbacher, 2024). Athletes’ superiority may be inspired by good performance in sport based on the capacity to regulate emotionality (for example, by means of attenuated self-talk) and move consistently with economized movements, exhibiting intended behavioral acts even during the pressure of competition (Hatfield et al., 2020). Athletic superiority is founded on the demonstration of athletic skills and the achievement of goals and superior formal results (Hämäläinen, 2013). Some beliefs and stereotypes about the variations in people’s abilities could also explain perceived athletic superiority (L. Harrison et al., 2007; L. Harrison et al., 2019; Kerr, 2010).

Athletes’ feelings of superiority could also be due to qualitative superiority (the ability of an individual or the team to be better than the competitors), numerical superiority (more players from the team in a given area of the field), positional superiority (positioning players from the team in areas of the field that give an advantage to the team), dynamic superiority (time and speed of movements that give an advantage to a player), and cooperative superiority (good relationships in the team that create group cohesion and positive psychological climate) (Motzenbecker, 2020). Successful expert athletes also feature cognitive superiority of their good attention and memory, as well as automaticity of skills (Chu & Wang, 2024). More strongly expressed feelings of superiority and higher self-esteem among athletes compared to non-athletes may adequately reflect mastery of skills and good physical form achieved by means of regular sports training.

In support of our findings about a positive correlation between the years of sport activity and self-esteem, some other studies found that self-esteem was significantly higher among those practicing sport for 10 years and more compared to those practicing sport for less than ten years (Uçan & Çağlayan, 2012), as well as that the most experienced athletes had significantly higher levels of self-esteem and athletic identity than the least experienced athletes (Maher, 2016). High self-esteem is necessary for athletes to be confident in their ability to perform well during sports competitions.

Regarding the comparison between the athletes practicing individual or team sports, in support of our findings, some other authors (Buhril, 2019; Maher, 2016) also report the lack of statistically significant differences in self-esteem between the athletes practicing individual or team sports, but they studied only female athletes with a State Self-Esteem Scale (Buhril, 2019) or only male athletes differing in their sports experience with the Rosenberg Self-Esteem Scale (Maher, 2016). Some other studies report a higher level of self-esteem (measured with the Rosenberg Self-Esteem Scale) among experienced male athletes practicing individual sports for 10–15 years compared to experienced male athletes practicing team sports for 10–15 years (Šagát et al., 2021) or a higher level of personal perceived self-esteem but not significant differences in social perceived self-esteem (measured with a self-esteem inventory) among male athletes practicing team sports compared to the male athletes practicing individual sports (Singh & Battan, 2019). A lack of statistically significant differences in global self-esteem has been found between athletes practicing non-contact, contact, and collision sports (Sanader et al., 2021). It seems that the differences between individual and team sportspeople in self-esteem could depend on the aspect of self-esteem that is studied—global or partial self-esteem—the period of sport practice, cultural belonging, and possibly gender belonging. There are some significant differences in the inferiority feelings among four countries with the highest scores in Malaysia and India (Čekrlija et al., 2020). Some significant differences have been found in general self-esteem among Turkish and Montenegrin teenage basketball players that could be due to the cultural values (Sari et al., 2013). In the scientific literature, the findings regarding gender differences in self-esteem are contradictory—some authors do not report any significant gender differences in self-esteem (Dilova et al., 2017; Jain & Dixit, 2014); some other authors report a higher self-esteem in men (Bleidorn et al., 2015; Minev, 2018) or a higher self-esteem in women (Papazova, 2010) measured with different questionnaires. Women are more likely to exaggerate positive self-descriptions, and men are more likely to deny negative self-descriptions (He et al., 2015), so both genders differ in the aspects of self-esteem on which they emphasize (Stoyanova et al., 2020).

The scientific literature reports the lack of any significant gender differences in superiority specifically among athletes (Kondo et al., 2022), as well as the lack of gender differences in inferiority feelings among people with different occupations (Čekrlija et al., 2020; Kolisnyk et al., 2020) and lack of gender differences in superiority feelings among people with different occupations (Kolisnyk et al., 2020). Our findings about gender differences in inferiority feelings, superiority feelings, and self-esteem are presented in Appendix B, and they suggest that the differences in inferiority feelings, superiority feelings, and self-esteem could be better explained by means of practicing sport or not than by gender differences.

Our findings support the established trends that physical exercises negatively predict inferiority feelings (T. Yang et al., 2023), sport activity is related to reduced inferiority complex (Liu, 2022), inferiority feelings accompany poor performance in sports (Hirao, 2014), while self-esteem is enhanced during physical activity (Liu, 2022). Sport activity seems to have the potential to reduce inferiority feelings and improve self-esteem and superiority feelings in the process of social comparison. Physical exercises reduce the feelings of inferiority directly, as well as indirectly, mediated by athletes’ good emotional regulation and social support received by family, friends, teams, and sport fans (Peng et al., 2025). Practicing sport facilitates control over one’s own body and self-confidence (Adler, 2008).

The inferiority feelings for the non-athletes might be related to poor performance, dissatisfaction with bodily attractiveness, and unsatisfactory social skills (Kosaka, 2008) compared with older and more experienced colleagues. The feelings of inferiority may be due to low autonomy, restriction of freedom and overprotection, or neglection and rejection (Adler, 2002; Ferguson, 2016) that seem more probable for non-athletes compared to athletes who put independent efforts to improve and succeed rewarded by social recognition.

Some other authors have found a weak but statistically significant negative correlation between age and the inferiority complex (Kolisnyk et al., 2020), as well as between age and self-esteem (Brailovskaia & Margraf, 2020). The studied athletes and non-athletes were similar in their ages; the non-athletes were slightly older. That is why the non-athletes’ higher inferiority feelings and lower self-esteem should not be due to age peculiarities.

Self-esteem is higher among working people than among non-workers (Gergov, 2024), and sport is a way of self-realization for athletes that also may explain their higher self-esteem compared with the studied non-athletes.

Self-esteem correlates positively with motivation for affiliation (Mavrodiev & Gergov, 2021), and team sports give the opportunity to satisfy the need for affiliation in a socially valued way that may explain higher self-esteem among those practicing team sports.

It seems that practicing sport is a protective factor for personal well-being, as higher self-esteem (more characteristic for the athletes, according to our findings) is positively related to life satisfaction and happiness, while low self-esteem (more characteristic for non-athletes, according to our results) is a predisposition for depression and lack of efficient coping strategies (Brailovskaia & Margraf, 2020).

The limitations of this study are related to sample size, as our sample could reveal some trends but cannot be representative of all athletes and non-athletes. The participants’ scores may be influenced by social desirability and cultural peculiarities, because another study has found some significant differences in the inferiority feelings between four countries (Čekrlija et al., 2020) or educational differences, as people with higher educational levels have less intense inferiority feelings compared with people with low educational levels who have inferiority complex propensity (Kolisnyk et al., 2020). The athletes in our sample were not better educated than the non-athletes; they had just different types of education.

The studied non-athletes had studied physical education at school, and they may have practiced sport sporadically or may have casually participated in sports events, so considering them with 0 sports experience could lead to a loss of valuable data about varying levels of sport participation for non-athletes and even artificial inflation of group differences. However, answering the question “How long have you been practicing sport?”, the non-athletes reported that they did not practice sport. Correspondingly, the non-athletes also did not indicate any type of sport practiced by them. That was among the reasons for coding a non-athlete’s sports experience with 0. Of course, it would be difficult for the individuals to estimate the length of their own sport experience if it was only casual engagement in leisure physical activities. It seems that casual sport activity is considered as not enough for a person to identify oneself with sport, but it would be valuable to establish how casual sport activity could be related to inferiority feelings, superiority feelings, and self-esteem, so this could be considered among the limitations of the study. The years of studying physical education at school were not considered as sport experience because their number was the same for all participants who had graduated from at least secondary education, and the participating non-athletes did not report them as an experience of practicing sport. For the goals of this study, an athlete was considered an individual who was a member of a sport club and/or studied a sport major in a university or had a document of sport qualification and/or practiced sport systematically, i.e., at least three times per week (Liutsko et al., 2024) since a minimum of one year (Till et al., 2022).

Another limitation of this study was focusing only on the sport experience as related to self-esteem, inferiority feelings, and superiority feelings, and not asking the participating athletes about their achievements and success in sport. Sport achievements are an important factor for athletes’ self-esteem. It has been found in the scientific literature that elite athletes had higher self-esteem than non-elite athletes (Petrovska et al., 2022), national athletes had higher self-esteem than regional athletes (Saha et al., 2024), and successful athletes who gained medals in regional, national, and international competitions had higher self-esteem than non-successful athletes (Matmask & Ozrudi, 2019). However, self-esteem and achievement motivation correlated positively and moderately for both successful and unsuccessful athletes (Matmask & Ozrudi, 2019). Motivation for achievement could be considered as a striving for acquiring and manifesting higher ability than the competitors or one’s previous level of mastery and performance (Saha et al., 2024). This definition relates motivation for achievement to the feelings of superiority. A longer period of sport training may express higher motivation for practicing sport and consequently, improving own mastery and performance. Years of sports training may be an important correlate of self-esteem, because it has been found that the students who participated in sport prior to their enrollment in a university or college had higher self-esteem and were happier than the students who did not practice sport prior to their university studies (Collins, 2018).

## 5. Conclusions

To the best of our knowledge, this was the first study to compare inferiority and superiority feelings between athletes and non-athletes in connection with their self-esteem. In line with the scientific findings up to these moments, it has been found that the athletes (practicing individual or team sports) had higher self-esteem and more strongly expressed feelings of superiority, as well as less experienced feelings of inferiority than the non-athletes. A lower degree of inferiority feelings, even for a younger age, was the characteristic that mainly distinguished the studied athletes from the non-athletes. Practicing sport seems to be a source of self-confidence and higher self-esteem based on positive self-evaluations of one’s own health status, physical functioning, increased power, velocity, and endurance; improved visual–motor coordination; achievements; and social recognition that might contribute to diminishing inferiority feelings, increasing superiority feelings, and other positive emotions such as pride and happiness.

The study of self-esteem, feelings of inferiority, and superiority among athletes and non-athletes could have some practical implications in various areas, revealing some factors for maintaining good mental health (weaker feelings of inferiority, higher self-esteem, and stronger feelings of superiority could be a protective factor against depression and anxiety improving well-being and strengthening mental resilience), supporting self-confidence and encouraging goals setting with the aim of good performance in both athletic and everyday life.

## Figures and Tables

**Table 1:** Descriptive parameters of the sample.

Statistics	Athletes	Non-Athletes
Number	197	198
Male (*N*)	98	99
Female (*N*)	99	99
Mean age	20.6	23.8
Standard deviation of age	2.8	6.1
Minimum age	18	18
Maximum age	38	38
Mean years of sport experience	8.3	0
Standard deviation of years of sport experience	4.9	0
Minimum years of sport experience	1	0
Maximum years of sport experience	30	0

Note: The years of studying physical education at school were not considered as sport experience. Their number was the same for all participants who had graduated from at least secondary education. The non-athletes reported that they did not practice any sport.

**Table 2:** Coefficients of skewness and kurtosis of the inferiority scale COMPIN-10, superiority scale SUCOMP-10, single-item self-esteem measure SISES, age and years of sport experience.

Statistics	COMPIN-10	SUCOMP-10	SISES	Age	Years of Sport Experience
Skewness	0.572	−0.148	−0.721	1.916	1.097
Standard error of skewness	0.123	0.123	0.123	0.123	0.123
Kurtosis	−0.381	−0.358	0.172	2.539	0.502
Standard error of kurtosis	0.245	0.245	0.245	0.245	0.245

**Table 3:** Differences between the non-athletes and the athletes practicing individual and team sports in their inferiority feelings, superiority feelings, and self-esteem.

Variables	Groups	*N*	Mean	Standard Deviation	Test of Homogeneity of Variances	Anova	Welch	Effect Size Cohen’s *f*
Inferiority feelings	Non-athletes	198	2.59	0.91	Levene_(2, 392)_ = 5.701, *p* = 0.004	*F*_(2, 392)_ = 23.069, *p* < 0.001	Welch_(2, 186.599)_ = 23.354, *p* < 0.001	0.290, i.e., medium effect size
	Individual sport	128	2.00	0.72				
	Team sport	69	2.07	0.79				
Superiority feelings	Non-athletes	198	3.03	0.71	Levene_(2, 392)_ = 0.257, *p* = 0.774	*F*_(2, 392)_ = 3.598, *p* = 0.028	Welch_(2, 183.240)_ = 3.711, *p* = 0.026	0.119, i.e., small effect size
	Individual sport	128	3.21	0.77				
	Team sport	69	3.24	0.66				
Self-esteem	Non-athletes	198	4.44	1.60	Levene_(2, 392)_ = 5.594, *p* = 0.004	*F*_(2, 392)_ = 5.822, *p* = 0.003	Welch_(2, 187.729)_ = 5.848, *p* = 0.003	0.169, i.e., small effect size
	Individual sport	128	4.88	1.38				
	Team sport	69	5.06	1.39				

Note: Effect size Cohen’s f was computed by means of the procedure offered by Lenhard and Lenhard (2022), and it was interpreted according to Cohen (1988, pp. 285–287).

## Data Availability

The data that support the findings of this study are available from the first author, upon reasonable request.

## Appendix A. The Questionnaires in Bulgarian

Въпросник COMPIN-10

Инструкция: С помощта на този въпросник се изследва склонността към самоподценяване. Всяко от следващите твърдения се отнася до Вас самия. Към всяко твърдение се предлагат 5 възможни отговора. От тях изберете този, който Ви подхожда най-много и го оградете.

**Твърдения**	**1 Изобщо не съм съгласен/а**	**2 По-скоро не съм съгласен/а**	**3 Не мога да преценя, колкото съм съгласен/а, толкова и несъгласен/а**	**4 По-скоро съм съгласен/а**	**5 Напълно съм съгласен/а**
1. Не знам как да използвам компетенциите си в точния момент.	1	2	3	4	5
2. Знам, че се подценявам, но не мога да се справя с това.	1	2	3	4	5
3. Когато работя с други, изглежда, че не се справям толкова добре, колкото те.	1	2	3	4	5
4. Не мога да изразявам себе си и да задържа хората, които обичам, до себе си.	1	2	3	4	5
5. По време на работа си повтарям: няма да успея, така че щеше да е по-добре, ако изобщо не се бях захващал/а	1	2	3	4	5
6. Често усещам, че няма да мога да направя това, което се очаква.	1	2	3	4	5
7. Лесно ме възпират неуспехите и ми е трудно да продължа.	1	2	3	4	5
8. Често чувствам, че не съм готов/а за неща, които трябва да направя.	1	2	3	4	5
9. Не се уважавам достатъчно.	1	2	3	4	5
10. Не съм уверен/а в себе си.	1	2	3	4	5

Въпросник SUCOMP-10

Инструкция: С помощта на този въпросник се изследва чувството за превъзходство. Всяко от следващите твърдения се отнася до Вас самия. Към всяко твърдение се предлагат 5 възможни отговора. От тях изберете този, който Ви подхожда най-много и го оградете.

**Твърдение**	**1 Изобщо не съм съгласен/а**	**2 По-скоро не съм съгласен/а**	**3 Не мога да преценя, колкото съм съгласен/а, толкова и несъгласен/а**	**4 По-скоро съм съгласен/а**	**5 Напълно съм съгласен/а**
1. Когато правя нещо, за мен е важно да съм най-добрият и в повечето случаи успявам да бъда.	1	2	3	4	5
2. Начинът ми на мислене е много оригинален.	1	2	3	4	5
3. Мога да издържам и да работя повече, отколкото мнозинството хора.	1	2	3	4	5
4. Много малко хора са успявали толкова, колкото мен.	1	2	3	4	5
5. Много малко хора могат да се сравняват с мен.	1	2	3	4	5
6. Това, което за мен е нещо обикновено, много хора биха го считали за успех.	1	2	3	4	5
7. Обикновено не може да се намери решение без мен.	1	2	3	4	5
8. Понякога не изпълнявам собствените си очаквания, но знам, че другите не биха постигнали дори толкова.	1	2	3	4	5
9. Най-лошото нещо би било, ако нямаше хора като мен.	1	2	3	4	5
10. Много неща ме интересуват и мисля, че това ме прави различен/а от другите хора.	1	2	3	4	5

Въпросник SISES

Моля, оградете този отговор за следващото твърдение, който в най-голяма степен се отнася за Вас.

**Аз имам висока себеоценка.**						
①	②	③	④	⑤	⑥	⑦
**Не съвсем вярно за мен**						**Много вярно за мен**

## Appendix B. Results Regarding Gender Differences in Self-Esteem and Inferiority and Superiority Feelings

There were not any significant differences between the male and female participants in their self-esteem (*t*_(393)_ = 0.242, *p* = 0.809), neither between the male and female non-athletes in their self-esteem (*t*_(196)_ = 0.044, *p* = 0.965), nor between the male and female athletes in their self-esteem (*t*_(195)_ = 0.414, *p* = 0.679). The female athletes (*N* = 99; *M* = 4.98; *SD* = 1.38) had significantly higher self-esteem (*t*_(190)_ = 2.515, *p* = 0.013; *F*_Levene_ = 7.366, *p*_Levene_ = 0.007) than the female non-athletes (*N* = 99; *M* = 4.43; *SD* = 1.65). The male athletes (*N* = 98; *M* = 4.90; *SD* = 1.39) had significantly higher self-esteem (*t*_(192.8)_ = 2.156, *p* = 0.032; *F*_Levene_ = 4.450, *p*_Levene_ = 0.036) than the male non-athletes (*N* = 99; *M* = 4.44; *SD* = 1.56).

There were not any significant differences between the male and female participants in their inferiority feelings (*F*_Levene_ = 5.137, *p*_Levene_ = 0.024; *t*_(386.168)_ = 1.767, *p* = 0.078), nor between the male and female non-athletes in their inferiority feelings (*t*_(196)_ = 0.241, *p* = 0.810). The female athletes (*N* = 99; *M* = 2.20; *SD* = 0.73) had significantly higher inferiority feelings (*t*_(195)_ = 3.360, *p* = 0.001) than the male athletes (*N* = 98; *M* = 1.85; *SD* = 0.72). There was an interaction effect of gender and practicing sport/not practicing sport on the inferiority feelings (*F*_(1, 394)_ = 5.181, *p* = 0.023). The female athletes (*N* = 99; *M* = 2.20; *SD* = 0.73) and the female non-athletes (*N* = 99; *M* = 2.58; *SD* = 0.86) differed statistically significant in their inferiority feelings (*F*_(1, 391)_ = 10.472, *p* = 0.001) and the female non-athletes had significantly more intensive inferiority feelings than the female athletes (*p*_Bonferroni_ = 0.001). The male athletes (*N* = 98; *M* = 1.85; *SD* = 0.72) and the male non-athletes (*N* = 99; *M* = 2.61; *SD* = 0.97) differed statistically significant in their inferiority feelings (*F*_(1, 391)_ = 41.508, *p* < 0.001) and the male non-athletes had significantly more intensive inferiority feelings than the male athletes (*p*_Bonferroni_ < 0.001).

There were not any significant differences between the male and female non-athletes in their superiority feelings (*t*_(196)_ = 1.079, *p* = 0.282). The female athletes (*N* = 99; *M* = 3.35; *SD* = 0.64) had significantly higher superiority feelings (*F*_Levene_ = 4.025, *p*_Levene_ = 0.046; *t*_(186.617)_ = 2.513, *p* = 0.013, Cohen’s *d* = 0.358, i.e., small effect size, according to Lenhard and Lenhard (2022)) than the male athletes (*N* = 98; *M* = 3.09; *SD* = 0.79). The female athletes (*N* = 99; *M* = 3.35; *SD* = 0.64) had significantly higher superiority feelings (*t*_(196)_ = 2.783, *p* = 0.006) than the female non-athletes (*N* = 99; *M* = 3.08; *SD* = 0.71). There was a trend the male athletes (*N* = 98; *M* = 3.09; *SD* = 0.79) to have higher superiority feelings than the male non-athletes (*N* = 99; *M* = 2.97; *SD* = 0.72), but this difference was not statistically significant (*t*_(195)_ = 1.098, *p* = 0.273).

## References

[B1-ejihpe-15-00022] Adeka P. (2019). The concept of inferiority and superiority complex.

[B2-ejihpe-15-00022] Adler A. (2002). Individualna psihologia: Praktika i teoria *[Individual psychology: Practice and theory]*.

[B3-ejihpe-15-00022] Adler A. (2007). Chovekoznanie *[Understanding human nature]*.

[B4-ejihpe-15-00022] Adler A. (2008). Vazpitanie na detsata *[Child’s education]*.

[B5-ejihpe-15-00022] Anastasiei B. (2015). SPSS data analysis made easy. Become an expert in advanced statistical analysis with SPSS *[Video]*.

[B6-ejihpe-15-00022] Bleidorn W., Arslan R. C., Denissen J. J. A., Rentfrow P. J., Gebauer J. E., Potter J., Gosling S. D. (2015). Age and gender differences in self-esteem-a cross-cultural window. Journal of Personality and Social Psychology.

[B7-ejihpe-15-00022] Brailovskaia J., Margraf J. (2020). How to measure self-esteem with one item? Validation of the German Single-Item Self-Esteem Scale (G-SISE). Current Psychology: A Journal for Diverse Perspectives on Diverse Psychological Issues.

[B8-ejihpe-15-00022] Buhril L. (2019). A comparative study of self-esteem between individual and team sports female players. International Journal of Yogic, Human Movement and Sports Sciences.

[B9-ejihpe-15-00022] Chu X.-Y., Wang Z.-J. (2024). Cognitive superiority of athletic sports expert and its formation mechanisms: A perspective from automaticity and abstraction. Advances in Psychological Science.

[B10-ejihpe-15-00022] Cohen J. (1988). Statistical power analysis for the behavioral sciences.

[B11-ejihpe-15-00022] Collins N. M. (2018). Effects of early sport participation on self-esteem and happiness. The Sport Journal.

[B12-ejihpe-15-00022] Čekrlija Đ., Đurić D., Mirković B. (2017). Validation of Adlerian inferiority (COMPIN) and superiority (SUCOMP) complex shortened scales. Civitas.

[B13-ejihpe-15-00022] Čekrlija Đ., Rokvić N. M., Shereena N. M., Sern K., Vujaković L., Stamenković D., Radev M. T., Jovančević A. (2020). Preliminary psychometric properties of the Inferiority Complex Scale (COMPIN-10) in Bosnian-Herzegovinian, Serbian, Indian and Malaysian culture. 16th International conference “days of applied psychology 2020: Psychology in the world of science”, Niš, Serbia, September 25th–26th 2020.

[B14-ejihpe-15-00022] Diel K., Broeker L., Raab M., Hofmann W. (2021). Motivational and emotional effects of social comparison in sports. Psychology of Sport and Exercise.

[B15-ejihpe-15-00022] Dilova M., Papazova E., Koralov M. (2017). Bulgarian standardization of Morris Rosenberg’s Self-Esteem Scale. Psychological Thought.

[B16-ejihpe-15-00022] Ferguson E. D. (2016). Vavedenie v teoriata na individualnata psihologia *[Adlerian theory. An introduction]*.

[B17-ejihpe-15-00022] Gergov T. K., Kozhanov V. I., Yakovleva T. V., Kaymakova K. D. (2024). Trudovaya zanyatost’ i dokhody kak determinanty samootsenki v zrelom vozraste [Labour employment and income as determinants of self-esteem in adulthood]. International scientific and practical conference «Issues of science and education: New approaches and current studies», 29 February 2024, Cheboksary.

[B18-ejihpe-15-00022] Harrison L., Crooms B., Sales L., Dagkas S., Azzarito L., Hylton K. (2019). Challenging the stereotypical construction of black physical superiority and intellectual inferiority in sport. ‘Race’, youth sport, physical activity and health: Global perspectives.

[B19-ejihpe-15-00022] Harrison L., Azzarito L., Burden J. (2007). Perceptions of athletic superiority: A view from the other side. Race Ethnicity and Education.

[B20-ejihpe-15-00022] Hatfield B. D., Jaquess K. J., Oh H., Tenenbaum G., Eklund R. C. (2020). The cognitive and affective neuroscience of superior athletic performance. Handbook of sport psychology.

[B21-ejihpe-15-00022] Hämäläinen M. (2013). Three standards of athletic superiority. Journal of the Philosophy of Sport.

[B22-ejihpe-15-00022] He J., Van De Vijver F. J. R., Espinosa A. D., Abubakar A., Dimitrova R., Adams B. G., Aydinli A., Atitsogbe K., Alonso-Arbiol I., Bobowik M., Fischer R., Jordanov V., Mastrotheodoros S., Neto F., Ponizovsky Y. J., Reb J., Sim S., Sovet L., Stefenel D., Villieux A. (2015). Socially desirable responding: Enhancement and denial in 20 countries. Cross-Cultural Research.

[B23-ejihpe-15-00022] Hirao K. (2014). Comparison of feelings of inferiority among university students with autotelic, average, and nonautotelic personalities. North American Journal of Medical Sciences.

[B24-ejihpe-15-00022] Jain S., Dixit P. (2014). Self esteem: A gender based comparison and the causal factors reducing it among Indian youth. International Journal of Humanities and Social Science Invention.

[B25-ejihpe-15-00022] Kerr I. B. (2010). The myth of racial superiority in sports. The Hilltop Review.

[B26-ejihpe-15-00022] Kisyov K. (2021). Macrostructural distribution of the specific training tools for classic mountain running in a combined model of preparation for “mainly uphill” and “up and downhill” variants. Trakia Journal of Sciences.

[B27-ejihpe-15-00022] Kolisnyk L., Čekrlija Đ., Kaiagurka B. (2020). Peculiarities of superiority and inferiority complexes of Ukrainians. Mental Health: Global Challenges Journal.

[B28-ejihpe-15-00022] Kondo M., Tsuchiya H., Sugo T. (2022). The structure of trait pride in sports: Focusing on emotional episodes of university student-athletes. International Journal of Sport and Health Science.

[B29-ejihpe-15-00022] Kosaka Y. (2008). Developmental changes in inferiority feelings in adolescents and young adults: Important areas of the self. Japanese Journal of Educational Psychology.

[B30-ejihpe-15-00022] Lamberson K. A., Wester K. L. (2018). Feelings of inferiority: A first attempt to define the construct empirically. Journal of Individual Psychology.

[B31-ejihpe-15-00022] Lenhard W., Lenhard A. (2022). Computation of effect sizes.

[B32-ejihpe-15-00022] Liu C. (2022). Research on the influence of college students’ participation in sports activities on their sense of inferiority based on self-esteem and general self-efficacy. Frontiers in Psychology.

[B33-ejihpe-15-00022] Liutsko L., Leonov S., Pashenko A., Polikanova I. (2024). Is frequency of practice of different types of physical activity associated with health and a healthy lifestyle at different ages?. European Journal of Investigation in Health, Psychology and Education.

[B34-ejihpe-15-00022] Maderbacher A. (2024). Physical superiority in football. An analysis of the systematic development of conditional abilities and their challenges in Austria’s football youth academies. SportRXiv.

[B35-ejihpe-15-00022] Maher L. (2016). A comparative study of self-esteem, mental toughness and athletic identity in team and individual sports: Male athletes. Bachelor thesis.

[B36-ejihpe-15-00022] Matmask E. A., Ozrudi M. F. (2019). Survey of self-esteem among successful and unsuccessful student athletes and this relationship by achievement motivation. Asian Exercise and Sport Science Journal.

[B37-ejihpe-15-00022] Mavrodiev S., Gergov T., Petkova T. V., Chukov V. S. (2021). Self-esteem and motivation for affiliations with students from the humanities. 7th International e-conference on studies in humanities and social sciences: Conference proceedings, 28 June 2021, Belgrade.

[B38-ejihpe-15-00022] Minev M. A. (2018). Samoocenka i psihopatologichni simptomi v unosheska vazrast. Avtoreferat na disertatsionen trud za prisazhdane na obrazovatelna I nauchna stepen “doctor” [Selfesteem and psychopatologic symptoms in adolescence. Ph.D. thesis abstract.

[B39-ejihpe-15-00022] Motzenbecker P. (2020). The five superiorities.

[B40-ejihpe-15-00022] Papazova E. (2010). Determinants of gender-role attitudes, self-esteem and affective balance at adolescence. Psychological Research.

[B41-ejihpe-15-00022] Pegler A. J., Gregg A. P., Hart C. M. (2019). The Rosenberg and the rest: Meta-research on the measurement of self-esteem in personality and social psychology (2004–2015). PsyArXiv Preprints.

[B42-ejihpe-15-00022] Peng B., Chen W., Wang H., Yu T., Kong M. (2025). A study on the relationship between physical exercise and feelings of inferiority among college students: The chain mediating effect of social support and emotional regulation ability. Frontiers in Psychology.

[B43-ejihpe-15-00022] Petrovska T., Sova V., Voronova V., Khmelnitska I., Borysova O., Kurdybakha O. (2022). Features of self-esteem and level of ambition in athletes of different qualifications. Journal of Physical Education and Sport.

[B44-ejihpe-15-00022] Pimentel C. E., da Silva F. M. D. S. M., dos Santos J. L. F., Oliveira K. G., Freitas N. B. C., Couto R. N., de Sampaio Brito T. R. (2018). Single-Item Self-Esteem Scale: Brazilian adaptation and relationship with personality and prosocial behavior. Psico-USF, Bragança Paulista.

[B45-ejihpe-15-00022] Przepiórkowska D. (2016). Translation of questionnaires in cross-national social surveys: A niche with its own theoretical framework and methodology. Między Oryginałem a Przekładem.

[B46-ejihpe-15-00022] Robins R. W., Hendin H. M., Trzesniewski K. H. (2001). Measuring global self-esteem: Construct validation of a single-item measure and the Rosenberg Self-Esteem Scale. Personality and Social Psychology Bulletin.

[B47-ejihpe-15-00022] Rokvic N. (2020). Alexithymia, disgust and the inferiority/superiority complex: An exploratory study. Engrami.

[B48-ejihpe-15-00022] Saha S., Singh M. H., Karmakar D., Upadhyay K. (2024). A comparative study of self-esteem and achievement motivation among various levels of volleyball players. International Journal of Physical Education, Sports and Health.

[B49-ejihpe-15-00022] Saloniki E. C., Malley J., Burge P., Lu H., Batchelder L., Linnosmaa I., Trukeschitz B., Forder J. (2019). Comparing internet and face-to-face surveys as methods for eliciting preferences for social care-related quality of life: Evidence from England using the ASCOT service user measure. Quality Of Life Research: An International Journal of Quality of Life Aspects of Treatment, Care and Rehabilitation.

[B50-ejihpe-15-00022] Sample size calculator.

[B51-ejihpe-15-00022] Sanader A. A., Petrović J. R., Bačanac L., Ivković I., Petrović I., Knezević O. M. (2021). Competitive trait anxiety and general self-esteem of athletes according to the sport type and gender. Primenjena psihologija.

[B52-ejihpe-15-00022] Sari İ., Ilić J., Ljubojević M. (2013). The comparison of task and ego orientation and general self-esteem of Turkish and Montenegrin young basketball players. Kinesiology.

[B53-ejihpe-15-00022] Schoenfeld B. J., Ogborn D., Krieger J. W. (2016). Effects of resistance training frequency on measures of muscle hypertrophy: A systematic review and meta-analysis. Sports Medicine.

[B54-ejihpe-15-00022] Singh J., Battan K. K. (2019). Study on self-esteem among individual sports, team sports and e-sports. International Journal of Physiology, Nutrition and Physical Education.

[B55-ejihpe-15-00022] Stoyanova S., Garvanova M., Papazova E. (2020). Self-esteem, life satisfaction and values (Coopersmith self-esteem inventory for adults, Diener et al.’s satisfaction with life scale, and Schwartz value survey).

[B56-ejihpe-15-00022] Sweeney T. J. (2009). Adlerian counseling and psychotherapy: A practitioner’s approach.

[B57-ejihpe-15-00022] Šagát P., Bartik P., Lazić A., Tohănean D. I., Koronas V., Turcu I., Knjaz D., Alexe C. I., Curițianu I. M. (2021). Self-esteem, individual versus team sports. International Journal of Environmental Research and Public Health.

[B58-ejihpe-15-00022] Till K., Lloyd R. S., McCormack S., Williams G., Baker J., Eisenmann J. C. (2022). Optimising long-term athletic development: An investigation of practitioners’ knowledge, adherence, practices and challenges. PLoS ONE.

[B59-ejihpe-15-00022] Uçan Y., Çağlayan N. (2012). Comparison of self-esteem scores of individual and team sport athletes and non-athletes. Nigde University Journal of Physical Education and Sport Sciences.

[B60-ejihpe-15-00022] World Medical Association (2024). WMA declaration of Helsinki—Ethical principles for medical research involving human subjects.

[B61-ejihpe-15-00022] Yang D., Qiu B., Jiang J., Xia Y., Li L., Li Y., Luo L., Liu X., Meng J. (2023). Development of inferiority-compensation scale among high school students. BMC Medical Education.

[B62-ejihpe-15-00022] Yang T., Xiao H., Fan X., Zeng W. (2023). Exploring the effects of physical exercise on inferiority feeling in children and adolescents with disabilities: A test of chain mediated effects of self-depletion and self-efficacy. Frontiers in Psychology.

